# FOXO1 inhibits osteoclastogenesis partially by antagnozing MYC

**DOI:** 10.1038/srep16835

**Published:** 2015-11-16

**Authors:** Peng Tan, Hanfeng Guan, Linka Xie, Baoguo Mi, Zhong Fang, Jing Li, Feng Li

**Affiliations:** 1 Department of Orthopaedic Surgery, Tongji Hospital, Tongji Medical College, Huazhong University of Science and Technology, Wuhan, Hubei, China; 2 Cancer Center of Union Hospital, Tongji Medical College, Huazhong University of Science and Technology, Wuhan, China

## Abstract

FOXO transcription factors especially FOXO1 have profound roles in bone development and remodeling. The regulation of cells of the osteoblast lineage by FOXOs is suggested to be stage-specific or context dependent. Intriguingly, recent studies on the role played by FOXOs in osteoclastogenesis reached different conclusion. Bartell *et al.* showed that FOXOs restrained osteoclastogenesis and bone resorption partially by upregulation of the H_2_O_2_-inactivating enzyme catalase. Wang *et al.* demonstrated that FOXO1 activated osteoclast formation. In the present study, we confirmed the results of Bartell *et al.* that FOXO1 expression was reduced upon stimulation of RANKL; FOXO1 inhibition promoted and FOXO1 activation repressed, osteoclast differentiation and activity; the inhibitory effect of FOXO1 on osteoclastogenesis was partially mediated by ROS since treatment with ROS scavengers cancelled the effect of FOXO1 inhibition on osteoclastogenesis. We further investigated the mechanisms responsible for repressed osteoclastogenesis by FOXO1. We found that FOXO1 inhibition modulated MAPKs, NF-κB and AP-1. Finally, we proved that the inhibitory effect of FOXO1 on osteoclast formation was partially mediated by MYC suppression by showing that MYC repression almost totally abrogated the effect of FOXO1 inhibition on osteoclastogenesis. To conclude, our study confirmed FOXO1 as a cell-autonomous inhibitor of osteoclastogenesis.

Bone development and remodeling is dependent on the balance between bone formation by osteoblasts and bone resorption by osteoclasts^1^. The osteoclasts are multi-nucleated cells originated from hematopoietic stem cells^2^. Their formation and function is strictly regulated mainly by macrophage-colony stimulating factor (M-CSF) and receptor activator of NF-κB (RANK) ligand (RANKL)^3^,^4^. M-CSF promotes the survival and proliferation of osteoclast precursors and mature osteoclasts by activating extracellular signal-regulated kinase (ERK) and PI3K/AKT. Moreover, M-CSF facilitates osteoclast differentiation by stimulation of RANK expression on osteoclast precursors. RANKL binds to RANK on osteoclast precursors and mature osteoclasts, leading to the recruitment of adaptor molecules including TRAF6. TRAF6 then activates multiple downstream signaling pathways, such as NF-κB, MAPKs (ERK, JNK and p38), PI3K/AKT, AP-1 transcription factor family and NFATc1, initiating osteoclast formation and activity^5^,^6^,^7^,^8^. In addition, RANKL induces the generation of reactive oxygen species (ROS), which activates pathways including AKT, MAPKs and NF-kB, thereby promoting osteoclastogenesis^5^.

FOXO1 belongs to the subgroup O of forkhead transcription factors (FOX), which share the highly conserved forkhead DNA-binding domain^9^. FOXOs play essential roles in development and tumorigenesis. FOXOs regulate transcription of multiple target genes, thereby influencing varieties of cellular processes, e.g., apoptosis (target genes: BIM, NOXA, TRAIL and etc.), cell cycle arrest (CDKN1B, CCND1), redox balance (SOD2 and catalase). FOXOs were also shown to reduce ROS production by inhibition of mitochondrial function through decreased MYC activity^10^,^11^,^12^. The activity of FOXOs are mainly regulated by the PI3K/AKT pathway. AKT, the effector protein of PI3K signaling, phosphorylates FOXO proteins and leads to their inactivation and nucleus exclusion^9^.

FOXOs have complex roles in differentiation and tumorigenesis. In the hematopoietic lineage, FOXOs enhance survival of hematopoietic stem cells, inhibit myeloid lineage expansion and promote lymphoid differentiation^13^. FOXOs promote the function of leukemia-initiating cells *in vivo* and deletion of FOXOs improves survival of animals with acute myeloid leukemia (AML)^14^; while FOXOs act as tumor suppressors in lymphomagenesis^15^,^16^,^17^. Recent advances highlighted profound influences of FOXOs especially FOXO1 on bone homeostasis^18^,^19^,^20^,^21^,^22^. The regulation of the osteoblast lineage cells by FOXOs was suggested to be stage-specific or context dependent: FOXOs promote maintenance and differentiation of early progenitors^22^; repress proliferation of committed osteoblast precursors^18^; and FOXO1 is vital for osteocytes survival^19^. Efforts on elucidating the role played by FOXOs in osteoclastogenesis yielded contradictory results^23^,^24^. Bartell *et al.* showed that FOXO proteins is downregulated during osteoclast differentiation, deletion of FOXOs in osteoclasts increases bone resorption and overexpression of FOXO3 in osteoclasts decreases bone resorption. They attributed these effects to the regulation of ROS scavengers by FOXOs^24^. Wang *et al.* demonstrated that RANKL induced FOXO1 expression during osteoclast differentiation; FOXO1 deletion or silencing reduced osteoclast formation and activity^23^. We set out to resolve these discrepancies and better understand the role of FOXO1 in osteoclastogenesis.

## Results

### FOXO1 expression during osteoclastogenesis

**Figure 1:**
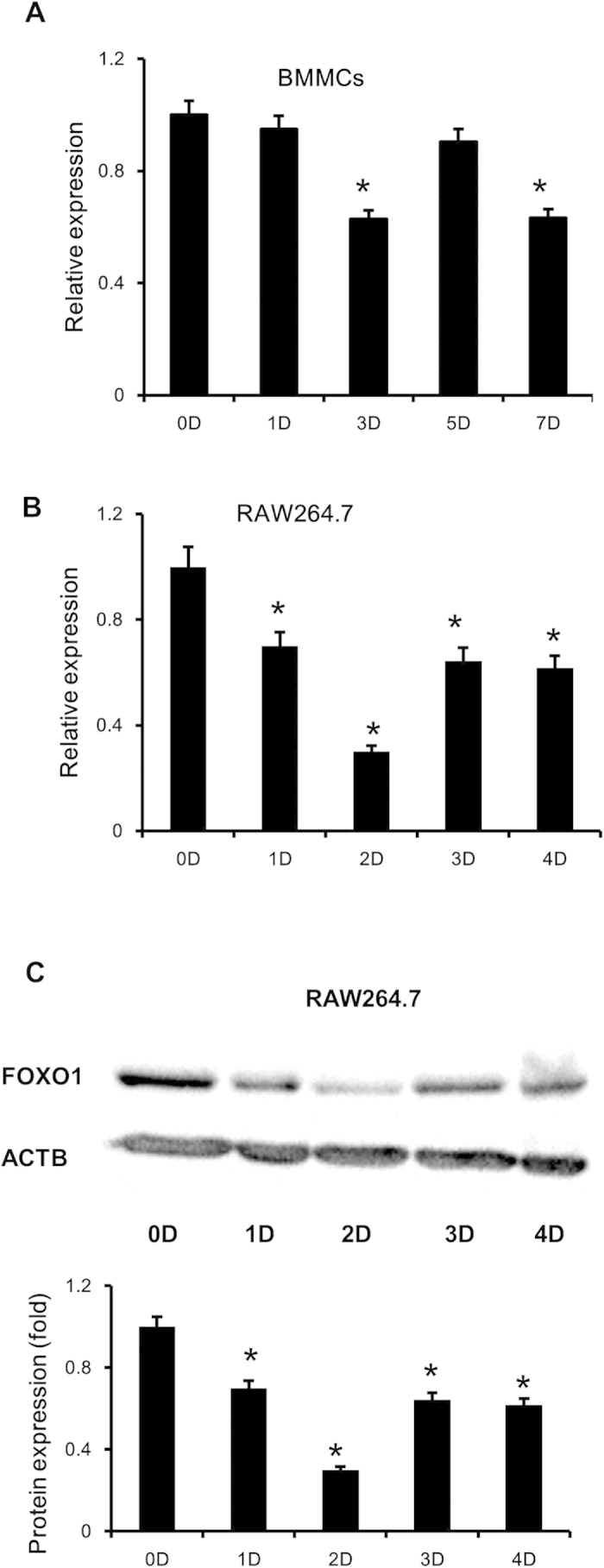
FOXO1 expression in RANKL stimulated BMMCs and RAW264.7 cells. (**A,B**) BMMCs were treated with M-CSF (30 ng/ml) and RANKL (50 ng/ml), RAW264.7 cells were treated with RANKL (50 ng/ml), the expression of FOXO1 was examined by real-time PCR. Data are mean ± SD of target gene to reference gene (FOXO1/β-actin) ratio. (**C,D**) Expression of FOXO1 protein in RAW264.7 cells incubated with RANKL (50 ng/ml) was assessed by Immunoblot. ACTB was used as a loading control. Band densities were quantified and normalized to the control. Data was presented as mean ± SD of 3 independent experiments. *P < 0.05 versus 0 day group.

We examined FOXO1 expression in BMMCs and RAW264.7 cells during osteoclastogenesis by quantitative real-time PCR. FOXO1 expression was decreased in BMMCs and RAW264.7 cells upon treatment of RANKL (Fig. 1A,B). The decrease was most significant at 3 days after RANKL incubation in BMMCs and 2 days in RAW264.7 cells. The expression of FOXO1 in osteoclastogenesis was also measured by Immunoblot in RAW264.7 cells. Similarly, RANKL decreased FOXO1 protein levels in RAW264.7 cells (Fig. 1C,D).

### Inhibition of FOXO1 promoted osteoclast differentiation and activity

**Figure 2:**
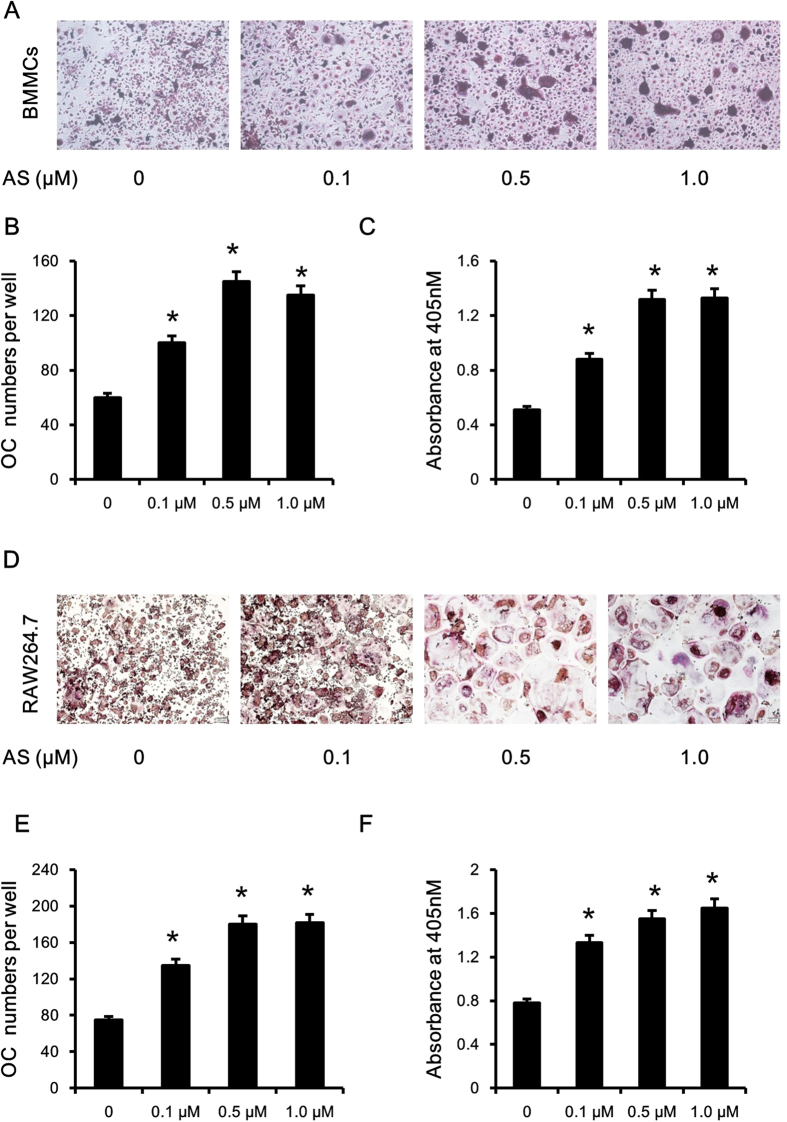
FOXO1 inhibition promoted osteoclast differentiation. (**A–C**) FOXO1 inhibition promoted osteoclastogenesis of mouse BMMCs. Mouse BMMCs were treated with the indicated doses of FOXO1 inhibitor (AS1842856) and M-CSF (30 ng/ml) and RANKL (50 ng/ml). After 7 days, cells were fixed for TRAP staining (**A**) and TRAP enzyme activity assay (**C**). The cells were photographed (original magnification, ×40; A) and the number of TRAP-positive multinucleated (> =3 nuclei) osteoclasts was counted (**B**). Data represent the mean±SD of 3 independent experiments. (**D–F**) FOXO1 inhibition promoted osteoclastogenesis of RAW264.7 cells. RAW264.7 cells were incubated with RANKL (50 ng/mL) and the indicated concentrations of AS1842856 for 4 days. TRAP staining and TRAP enzyme activity assay (**F**) were performed. The cells were photographed (**D**) and the number of TRAP-positive multinucleated osteoclasts was counted (**E**). Data represent the mean ± SD of 3 independent experiments. *P < 0.05 versus control group.

**Figure 3:**
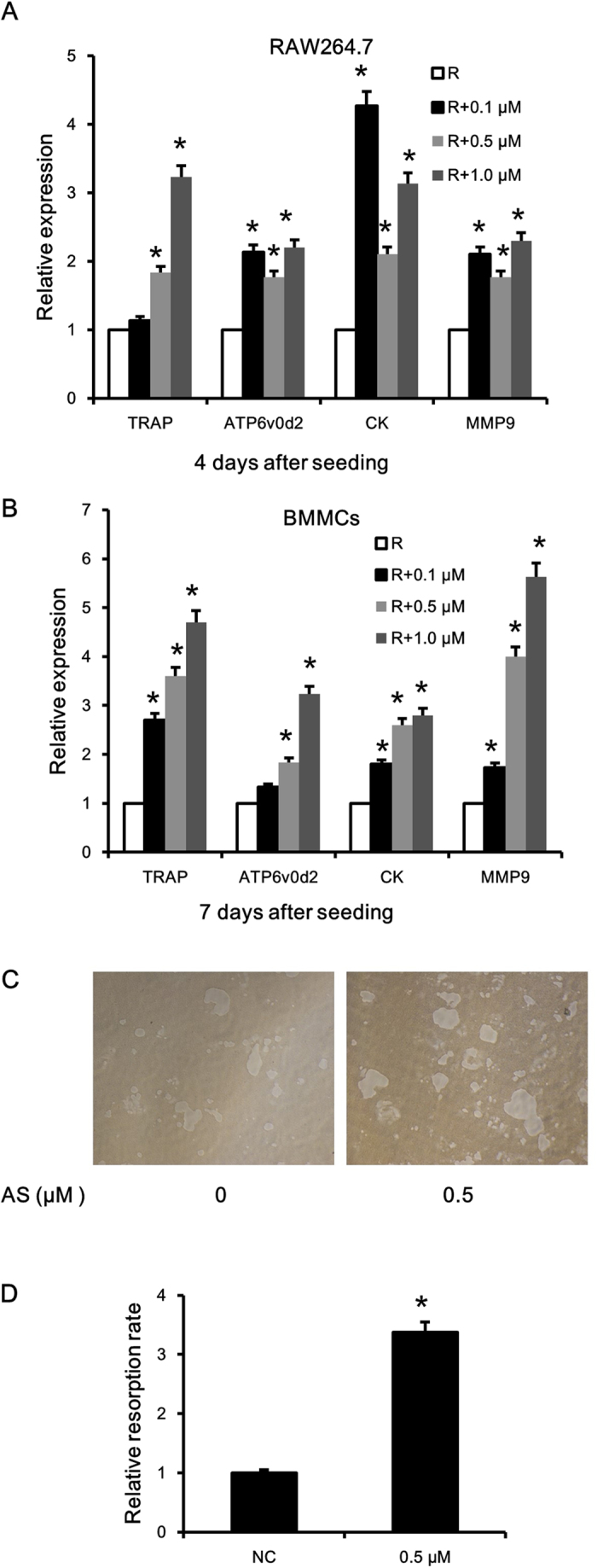
FOXO1 inhibition increased expression of osteoclast marker genes and promoted osteoclast function. RANKL-stimulated RAW 264.7 cells (**A**) and mouse BMMCs (**B**) were treated with the indicated doses of AS1842856. The expression of osteoclast marker genes (TRAP, ATP6v0d2, cathepsin K and MMP9) was examined by real-time PCR. The expression level was calibrated using theβ-actin house-keeping gene, and values indicating the fold-change to control are shown. The data shown are representative of three independent experiments performed in triplicate. *P < 0.05 versus treated with RANKL alone. (**C**) RAW264.7 cells were cultured with 50 ng/ml RANKL on a 6-well collagen pre-coated plate for 4 days. Formed osteoclasts were collected and seeded onto a Corning OsteoAssay Surface in a 96-wells plate, subsequently they were treated with 0.5 μM AS1842856 or vehicle in the presence of 50 ng/ml RANKL for 3 days. Then the disc was washed with 5% sodium hypochlorite for 5 min, and the resorption pits were visualized with light microscopy (original magnification, ×40). (**D**) The resorption area was analyzed with a Image-Pro Plus software. Three independent experiments were analyzed and the data are mean ± SD of FOXO1 inhibition group to vehicle group ratio.

To explore the roles played by FOXO1 in osteoclastogenesis, we used AS1842856, a specific inhibitor of FOXO1 transcription activity. We first measured the influence of AS1842856 on cell viability. AS1842856 had no significant influence on BMMCs proliferation and slightly promoted the proliferation of RAW264.7 cells (Supplemental Fig. 1). As shown in Fig. 2, treatment with AS1842856 significantly increased the number and size of TRAP-positive multinuclear cells in a dose-dependent manner in both BMMCs and RAW264.7 cells. Similarly, TRAP activity in culture medium also increased following AS1842856 treatment (Fig. 2C,F). The promotion of osteoclastogenesis was confirmed by the higher mRNA levels of TRAP, ATP6v0d2, CK, and MMP9 (Fig. 3A,B). To evaluate the potential influence of FOXO1 inhibition on the resorbing activity of osteoclasts, RAW264.7 cells were induced to differentiate into osteoclasts without AS1842856 treatment. Mature osteoclasts were collected, seeded onto a Corning OsteoAssay Surface and treated with AS1842856 (0.5 μM) in the presence of 50 ng/ml RANKL for 3 days. Then the resorption pits were visualized with light microscopy. As shown in Fig. 3C, FOXO1 inhibition obviously increased the resorption area. When quantified with a software, the increase of the resorption area was 3.4 fold (Fig. 3D).

### FOXO1 overexpression inhibited osteoclast differentiation and activity

**Figure 4:**
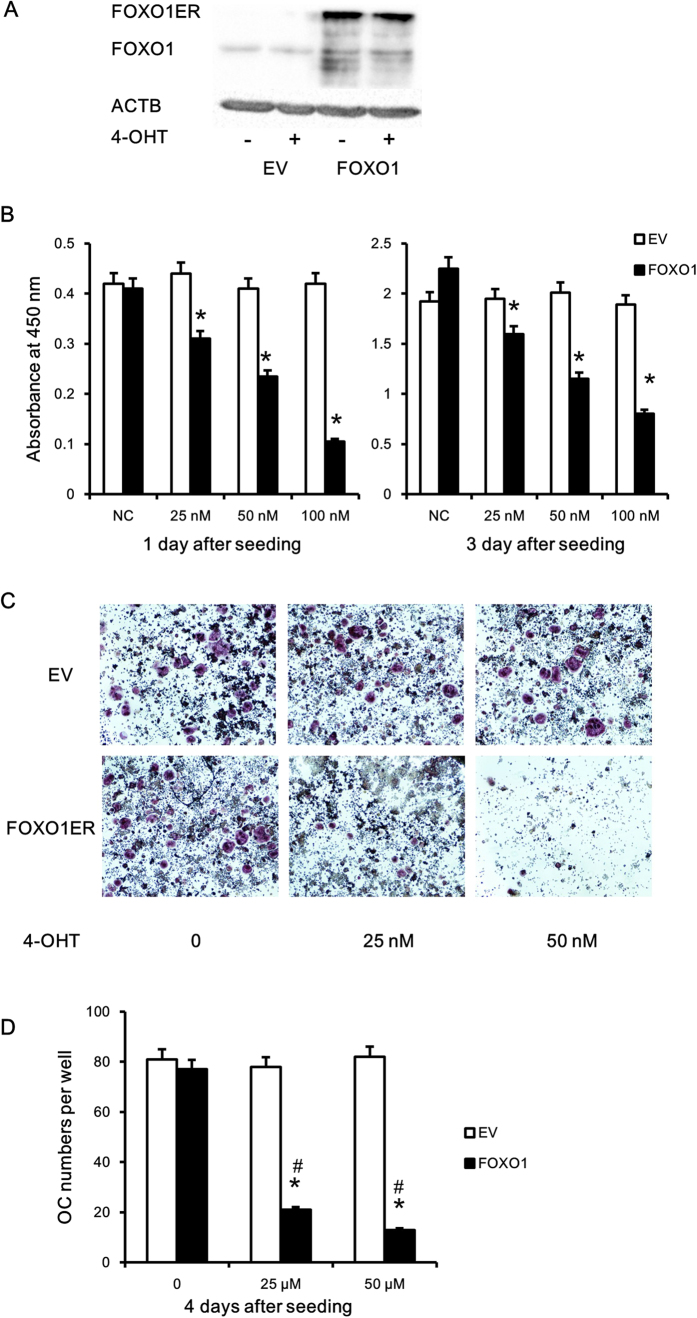
FOXO1 overexpression inhibited osteoclast differentiation. (**A**) Expression of FOXO1ER protein in stably infected RAW264.7 cells was validated by immunobloting using anti-FOXO1 antibody. Anti-ACTB antibody was used as a loading control. (**B**) RAW264.7 cells stably infected with empty vector or FOXO1ER were plated in 96-well plates at a density of 3 × 10^3^ cells per well (day 0). 4-OHT was added at the indicated doses and cell proliferation was detected by CCK8 colorimeter at day 1 and 3. (**C,D**) RAW264.7 FOXO1ER and RAW264.7 EV cells were seeded in 96-well plates at a density of 5 × 10^3^ cells per well, treated with 25 and 50 nM 4-OHT and stimulated with RANKL (50ng/ml) for 4 days. TRAP staining was performed and the cells were photographed (original magnification,×40; (**C**)), the number of TRAP-positive cells with three or more nuclei were counted under a microscope (**D**). Data represent the mean ± SD of 3 independent experiments. *P < 0.05 versus control group, ^#^P < 0.05 versus EV group.

**Figure 5:**
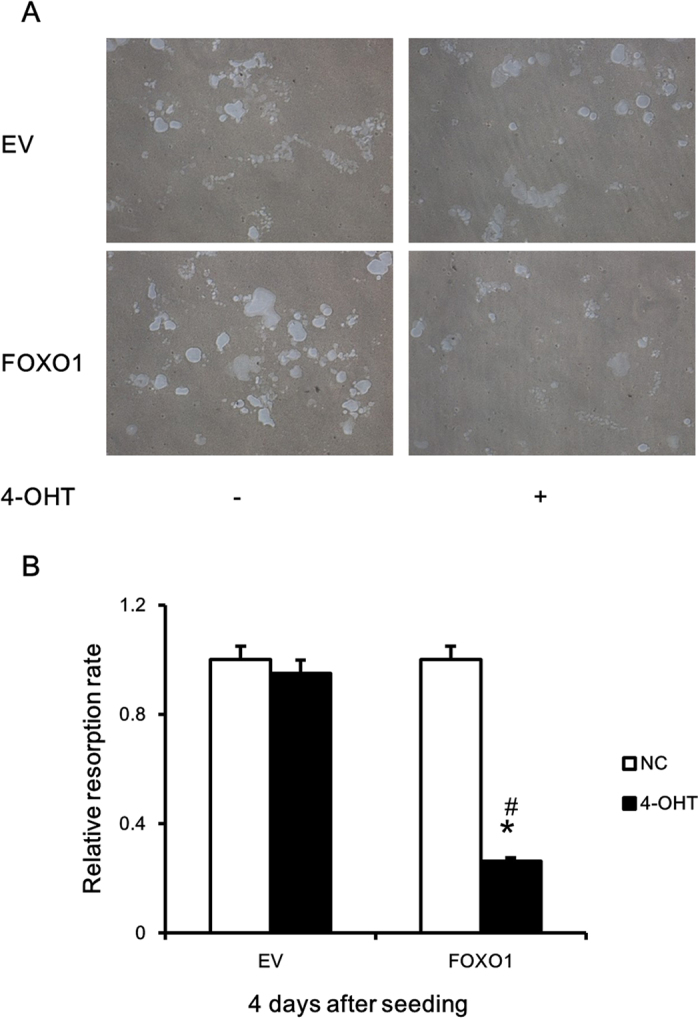
FOXO1 overexpression inhibited osteoclast function. (**A**) RAW264.7 FOXO1ER and RAW264.7 EV cells were cultured with 50 ng/ml RANKL on 6-well collagen pre-coated plates for 4 days. Formed osteoclasts were collected and re-seeded onto Corning OsteoAssay Surface (Corning Incorporated Life Science , USA) in a 96-wells plate, mature osteoclasts were then treated with 50 nM 4-OHT or vehicle in the presence of 50 ng/ml RANKL for 3 days. Then the resorption pits were visualized with light microscopy (original magnification, ×40). (**B**) The resorption area was analyzed with the Image-Pro Plus software. Three independent experiments were analyzed and data are mean ± SD of 4-OHT group to vehicle group ratio. *P < 0.05 versus control group, ^#^P < 0.05 versus EV group.

To validate the role played by FOXO1 in osteoclastogenesis, we overexpressed FOXO1 in RAW264.7 cells. We first verified the expression of FOXO1ER protein by immunoblotting (Fig. 4A). The infected cells showed strong expression of the fusion protein. When FOXO1ER was activated by 4-OHT, we observed a reduction of viable cells in raw264.7 cells in a dose-dependent manner (Fig. 4B). Furthermore, FOXO1ER activation decreased the number of TRAP + multi-nuclear osteoclast cells and TRAP activity (Fig. 4C,D). We then explored the effect of FOXO1 activity on osteoclast function. RAW264.7 FOXO1ER and RAW264.7 EV cells were allowed to differentiate into mature osteoclasts, reseeded onto a Corning OsteoAssay Surface, and treated with 4-OHT (50 nM) in the presence of RANKL for 3 days. Then the resorption pits were visualized and quantified. As shown in Fig. 5A, FOXO1ER activation remarkably decreased the bone resorption area of osteoclasts differentiated from RAW264.7 FOXO1ER cells. Compared to the control group, absorption area of the FOXO1ER activation group was reduced by about 3.8 time (Fig. 5B).

### FOXO1 inhibition modulated multiple pathways involved in osteoclastogenesis

**Figure 6:**
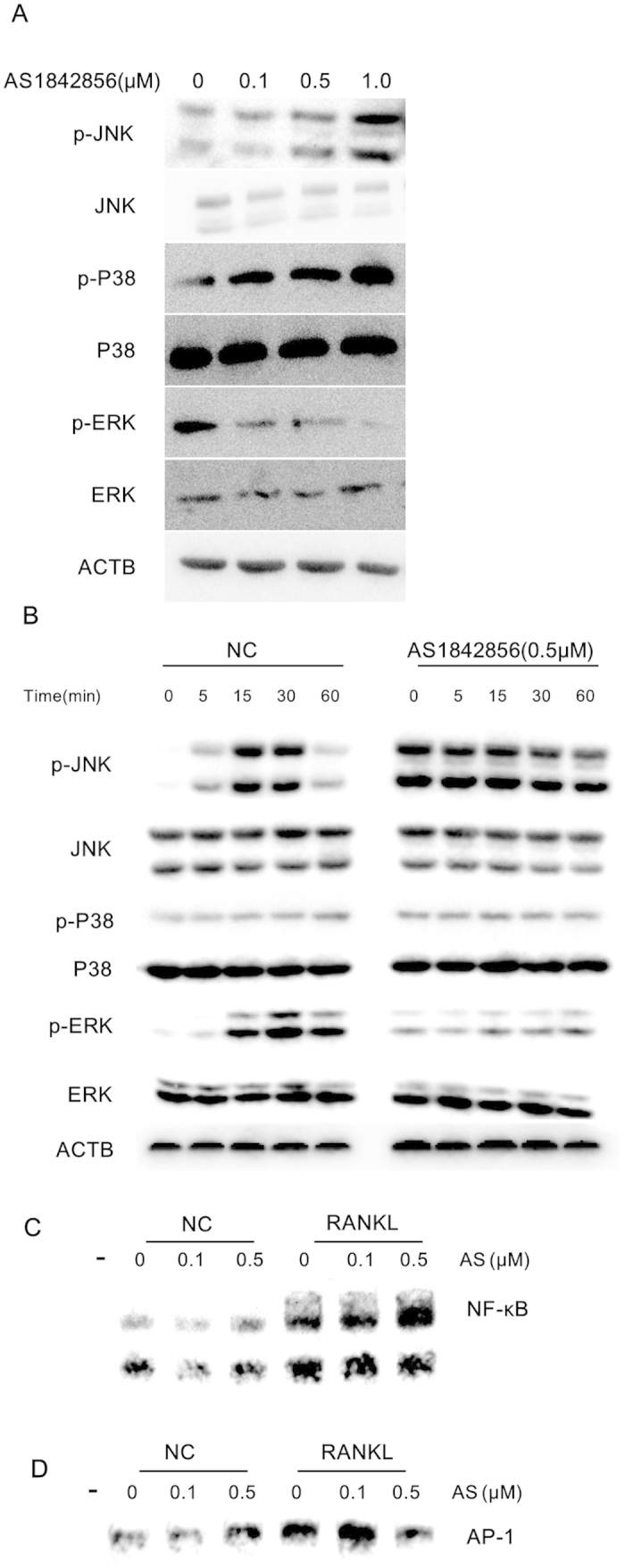
FOXO1 inhibition modulated multiple pathways involved in osteoclastogenesis. (**A**) Levels of p-JNK and p-P38 were increased by AS1842856 in RAW264.7 cells. After treatment with AS1842856 (at a concentration of 0.1, 0.5 or 1.0 μM) for 12 h, RAW264.7 cells were incubated with RANKL (50 ng/mL) for 30 min. Total proteins were then extracted for Western blot analysis. Antibodies to ACTB and total JNK, p38 and ERK served as loading controls. (**B**) After treatment with AS1842856 (0.5 μM) for 12 h, RAW264.7 cells were incubated with RANKL (50 ng/mL) for 5, 15, 30 and 60 min. Total proteins were then extracted and the expression of p-JNK, p-P38 and p-ERK were detected by Immunoblot. Antibodies to ACTB and total JNK, p38 and ERK were used as loading controls. (**C,D**) FOXO1 inhibition activated the transcriptional activity of NF-κB and AP-1. After treatment with 0.1 and 0.5 μM AS1842856 for 12 h, RAW264.7 cells were stimulated with RANKL (50 ng/mL) for 30 min. Nuclear extracts were prepared and analyzed for DNA binding activity of NF-κB (**C**) and AP-1 (**D**). Experiments were done at least 3 times. Representative images were shown.

We next investigated whether FOXO1 inhibition altered the signaling pathways stimulated by RANKL in RAW264.7 cells. AS1842856 treatment significantly increased phosphorylated JNK (p-JNK) and phosphorylated p38 (p-P38) levels in a dose-dependent manner, whereas phosphorylated ERK (p-ERK) decreased significantly (Fig. 6A,B). NF-κB and AP-1 transcription factor families play pivotal roles in osteoclastogenesis^25^,^26^. To further understand the signaling pathways mediated by FOXO1 in osteoclastogenesis, we examined whether FOXO1 inhibition influenced the DNA binding activity of NF-κB and AP-1. As shown in Fig. 6C,D, the DNA binding activity of NF-κB and AP-1 increased dramatically 30 min after stimulation with RANKL. AS1842856 (0.5 μM) treatment mildly increased the baseline NF-κB and AP-1 activity. Moreover, FOXO1 inhibition by AS1842856 significantly promoted the activation of NF- κB and AP-1 by RANKL.

### MYC inhibition abolished the promotion of osteoclastogenesis by FOXO1 inhibition

**Figure 7:**
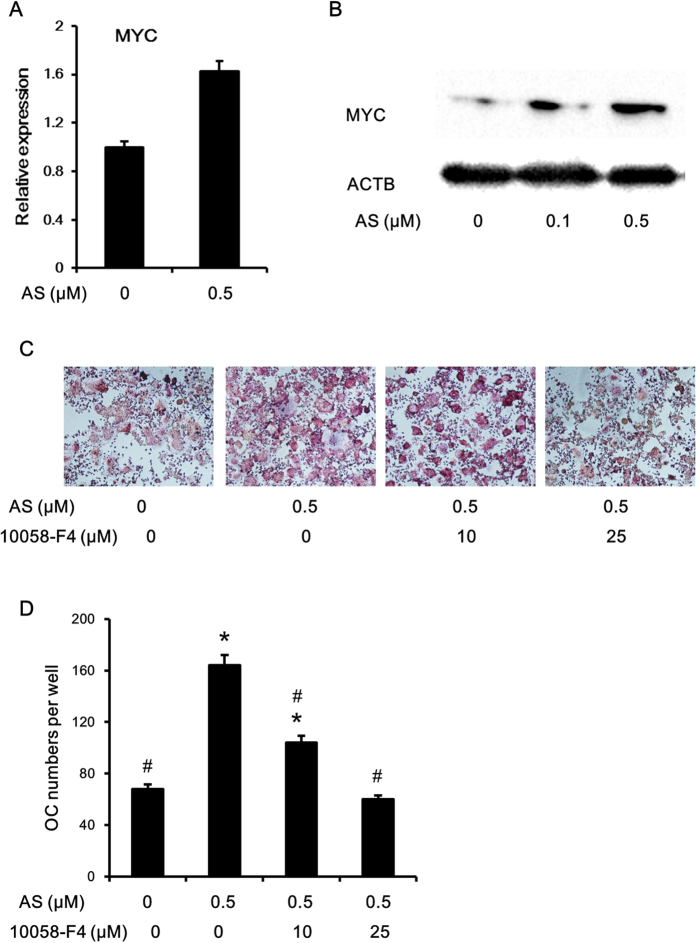
FOXO1 inhibition might promote osteoclastogenesis through upregulation of MYC expression. (**A,B**) RAW264.7 cells were treated with the indicated doses of AS1842856. 24 hours later, cells were harvested and the mRNA level of MYC was assessed by Q-PCR (**A**). Data represent the mean ± SD of 3 independent experiments. 48 hours after treatment, cells were collected and the protein level of MYC was detected by Immunoblotting (**B**). (**C,D**) RAW264.7 cells were incubated with RANKL (50 ng/mL), followed by treatment with the indicated doses of FOXO1 inhibitor (AS1842856) and MYC inhibitor (10058-F4). Four days later, cells were fixed for TRAP staining. The cells were photographed (original magnification,×40) and the numbers of TRAP-positive multinucleated (> = 3 nuclei) osteoclasts were counted (**E,F**). *P < 0.05 versus control group, ^#^P < 0.05 versus AS1842856 0.5 μM group.

MYC is a transcription factor involved in multiple cellular processes and is required for osteoclast differentiation^27^. We have recently shown that FOXO1 activation almost completely abolished MYC protein expression in lymphoma cells^17^. We speculated MYC might have mediated the effect of FOXO1 on osteoclastogenesis. MYC expression after FOXO1 inhibition was measured by Q-PCR and western blot, as shown in Fig. 7A,B, FOXO1 inhibition promoted the expression of MYC at the mRNA and especially the protein level. To further elucidate the role of MYC in FOXO1 mediated repression of osteoclastogenesis, we used 10058-F4, a small molecule inhibitor of MYC. RAW264.7 cells were treated with both AS1842856 and 10058-F4 in the presence of RANKL (50 ng/ml). 4 days later, cells were fixed for TRAP staining and images were taken. 10058-F4 abrogated the pro-osteoclastogenesis effect of FOXO1 inhibition in RAW264.7 cells (Fig. 7C,D) in a dose dependent manner.

## Discussion

In this study, we confirmed FOXO1 as an inhibitor of osteoclast differentiation and function and provided new insights into the mechanisms responsible. We found that FOXO1 expression was reduced upon initiation of osteoclastogenesis by treatment of RANKL. FOXO1 inhibition promoted and FOXO1 activation inhibited osteoclast differentiation and activity, respectively. The mechanisms could be attributed to modulation of MAPKs, NF-κB and AP-1. By showing that pretreatment with ROS scavengers (NAC, Supplemental Fig 2) or a MYC inhibitor cancelled the effect of FOXO1 inhibition, we proved that the inhibitory effect of FOXO1 on osteoclastogenesis was partially mediated by suppression of ROS generation and MYC.

Our results are in agreement with the previous study showing that the activity of FOXOs was suppressed via a RANKL/AKT axis, and FOXOs had a cell-autonomous inhibitory effect on osteoclastogenesis and survival of cells of the osteoclast lineage^24^. The previous study also focused on the reduction of ROS accumulation especially H_2_O_2_ by FOXOs. Our study, using a chemical inhibitor of FOXO1 transcription activity and a conditional overexpression system, confirmed the inhibitory effects of FOXO1 on osteoclast survival, differentiation and activity. We focused on the mechanisms of action of FOXO1 induced repression of osteoclast formation and activity. We found that FOXO1 inhibition activated JNK, P38, NF-κB and AP-1, and repressed ERK and MYC. These findings were in line with a previous study which found activated JNK and c-Jun (a member of the AP-1 transcription factor family) following FOXOs inhibition^14^. Reactive oxygen species (ROS), generated in response to RANKL stimulation, activates MAPKs and NF-kB^5^. As an established inhibitor of ROS levels, FOXO1 might repress MAPKs and NF-kB through ROS downregulation.

The regulation of osteoclast formation and resorption activity of the formed osteoclasts is complex. Some factors regulate both osteoclast formation and resorption activity of osteoclasts. For example, mutations in TNFSF11 (RANKL) and TNFRSF11A (RANK) lead to impaired osteoclast formation and resorption activity^28^. Other factors might regulate either formation or resorption activity. Mutations in TCIRG1, CLCN7, OSTM1, SNX10 and PLEKHM1 impair resorption activity, but not osteoclast formation; leading to the formation of abundant osteoclasts unable to resorb bone efficiently^28^. Several proteins in the apoptosis pathway including BCL-xL and BIM also have interesting effects on bone resorption by osteoclasts^29^,^30^. BIM induces apoptosis of osteoclast and leads to decreased number of osteoclast; however, BIM is a strong promotor of the bone-resorbing activity of osteoclasts. Therefore, bone resorption was increased despite the decreased number of osteoclasts with BIM overexpression^30^. Considering the complex regulation of cells of the hematopoietic lineage and osteoblast lineage by FOXOs, it might not be surprising that FOXO1 have complicated effects on osteoclast formation and resorbing activity of osteoclasts (for example, FOXO1 might repress osteoclast formation, whereas promotes the bone-resorbing activity of osteoclasts through transactivation of its target gene BIM^10^); leading to the disparate results acquired from the *in vivo* models generated with different approaches^23^,^24^.

Previous studies investigated the effects of FOXOs/FOXO1 on osteoclastogenesis and osteoclast resorbing activity using *in vivo* and *in vitro* experiments^23^,^24^. One limitation of the both studies might be that bone-resorbing activity of osteoclasts *per se* was not sufficiently assessed. All their data on bone resorption reflected the results of potential effects on osteoclast formation, survival and bone-resorbing activity of osteoclasts, and should be normalized to the number of osteoclasts. In our study, to investigate the influence of FOXO1 on bone-resorbing activity, we seeded equal number of mature osteoclasts, then we altered the activity of FOXO1 and measured bone resorption. We found that FOXO1 inhibition promoted and FOXO1 activation inhibited the resorption activity of osteoclasts, respectively. Our study confirmed that FOXO1 as a negative regulator of both osteoclast formation and resorption activity of osteoclasts.

We found that MYC might be an important factor mediating the effects of FOXO1 on osteoclastogenesis. FOXO3 was demonstrated as a negative regulator of mitochondrial function and ROS through inhibition of MYC^11^,^12^. We previously found that MYC inhibition led to upregulation of FOXO1 expression and forced FOXO1 expression inhibited mRNA and especially protein expression of MYC^17^. Our study, along with other studies^10^, suggested a mutual antagonism between FOXOs and MYC, not only in tumorigenesis, but also in cell differentiation.

In addition, FOXO1 activation in osteoblasts upregulated the expression of OPG^18^,^31^, the main anti-osteoclastogenic cytokine acting as a decoy receptor for RANKL. FOXOs triple knockdown in osteocytes led to increased number of osteoclasts^18^,^19^,^22^. These studies suggested that FOXOs might inhibit osteoclastogenesis via upregulation of OPG in cells of the osteoblast lineage. Our present study, along with a previous study^30^, proved FOXO1 as a cell-autonomous inhibitor of osteoclastogenesis. In conclusion, we believe that FOXO1 has cell-autonomous and -non-autonomous effects on osteoclastogenesis.

## Methods

### Cell culture and chemicals

Bone marrow mononuclear cells (BMMCs) were obtained from the femoral and tibial bone marrow of C57BL/6 mice at 6–8 weeks of age as described before^32^,^33^. The animal study was approved by the Institutional Animal Research Committee of Tongji Medical College. The method of the animal experiment was carried out in accordance with protocols approved by the Institutional Animal Care and Use Committee. BMMCs and RAW264.7, a murine monocytic cell line, were maintained in α-MEM supplemented with 10% heat-inactivated fetal bovine serum, M-CSF (30 ng/mL, for BMMCs), penicillin (100 U/mL) and streptomycin (100 μg/mL) at 37 °C in a humidified incubator with an atmosphere of 95% air plus 5% CO_2_. The FOXO1 inhibitor AS1842856 and MYC inhibitor 10058-F4 were described earlier^17^,^34^ and purchased from Calbiochem (Merck Millipore Billerica, MA, USA).

### Retroviral vectors and infection of RAW264.7

The pCFG5-IEGZ retroviral vectors was described earlier^35^. The construct pCFG5-FOXO1(A3)ER contains a FOXO1-ER fusion gene, a constitutively active form of FOXO1 fused in-frame with a modified estrogen receptor (ER) ligand-binding domain, has been described earlier^16^,^36^. The FOXO1-ER fusion protein is specifically activated by 4-hydroxytamoxifen (4-OHT, Calbiochem, Merck Millipore Billerica, MA, USA). The Platinum-A, a retroviral packaging cell line (Cell Biolabs, San Diego, USA), was cultured and transfected with retroviral vectors using Lipofectamine (Life Technologies, Thermo Fisher Scientific, Waltham, Massachusetts, USA) according to the manufacture’s protocol. Forty-eight and 72 hours after transfection, GFP expression was evaluated with a fluorescent microscopy, supernatants were collected and supplemented with polybrene (8 μg/mL), and used for infection of RAW264.7 cells. Infected cells were selected with 50 μg/mL Zeocin (Calbiochem). After about 2 weeks’ selection, the percentage of GFP expressing cells determined by flow cytometric analysis was above 95%.

### Cytotoxicity assay

For cytotoxicity assay, cells were seeded in 96-well plates at 3 × 10^3^ cells/well. After 24 h, cells were treated as indicated. A Cell Counting Kit-8 kit (CCK8, Beyotime, Jiangsu, China) was used to measure cytotoxicity at one day and three days after treatment.

### TRAP staining and TRAP enzyme activity assay

TRAP staining was done in cultured RAW264.7 and BMM cells with a TRAP staining kit (Sigma-Aldrich, Shanghai, China) as described earlier^32^,^33^. TRAP-positive cells with three or more nuclei were identified as osteoclasts. Cell images were taken using a digital camera attached to a Nikon ECLIPSE TE2000-S microscope (Nikon, Japan). TRAP enzyme activity was measured with an assay kit (Sigma-Aldrich) following the manufacturer's instructions. Briefly, cultured medium was collected from osteoclasts formed by BMMCs and RAW264.7 cells, respectively. TRAP enzyme activity was measured with a Synergy fluorescence plate reader at 405 nm on a colorimetric plate reader.

### Bone pit formation by osteoclasts

RAW264.7, RAW264.7 FOXO1-ER and RAW264.7 EV cells were treated with 50 ng/ml RANKL on 6-well collagen pre-coated plates for 4 days. Mature osteoclasts were collected using 2.5 mg/mL collagenase in dissociation buffer (Life technologies) and seeded onto a Corning OsteoAssay Surface (Corning Incorporated Life Science, NY, USA) in a multiple well plate. For RAW264.7 cells, mature osteoclasts were then treated with 0.5 μM AS1842856 or vehicle in the presence of 50 ng/ml RANKL for 3 days. For RAW264.7 FOXO1-ER and RAW264.7 EV cells, mature osteoclasts were treated with 100 nM 4-OHT or vehicle for 3 days. Then the disc was washed with 5% sodium hypochlorite for 5 min, images were taken and the resorption area was quantified by image analysis (Bioquant Image Analysis, Nashville, TN).

### Quantitative RT-PCR

Quantitative real-time polymerase chain reaction (qRT-PCR) was performed as described before^32^. Briefly, total RNA were isolated from BMMCs and RAW264.7 cells using TRIzol reagent (Invitrogen, Carlsbad, CA, USA) and first-strand cDNA was synthesized with MMLV reverse transcriptase (Promega, Madison, WI, USA). Templates were amplified using QuantiTect SYBR Green PCR Kit (QIAGEN) on the iCycler real time PCR instrument (BIO-RAD, CA, USA). The primers were synthesized by Invitrogen, sequences 5' to 3', sense and antisense, were as follows: FOXO1:TCTCCAGTCTGGGCAAGAGG and GCTGGTTCGAGGACGAAATG; TRAP: GATGCCAGCGACAAGAGGTT and CATACCAGGGGATGTTGCGAA; ATP6v0d2: AGTGCAGTGTGAGACCTTGG and TCTGCAGAGCTTCTTCCTCA; cathepsin K (CK): GAAGAAGACTCACCAGAAGCAG and TCCAGGTTATGGGCAGAGATT; matrix metalloproteinase-9 (MMP-9): CTGGACAGCCAGACACTAAAG and CTCGCGGCAAGTCTTCAGAG; MYC: GTGTCTGTGGAGAAGAGGCA and GCGTAGTTGTGCTGGTGAGT; β-actin: ATTTCTGAATGGCCCAGGT and CTGCCTCAACACCTCAACC.

### Immunoblot

Immunoblot was done as described earlier^37^. The following first antibodies were from Cell Signaling Technology (Boston, MA, USA): FOXO1, phospho-p38 (Thr180/Tyr182), p38, phospho-JNK (Thr183/Tyr185), JNK, phospho-ERK1/2 (Thr202/Tyr204), ERK and MYC. The antibody against beta-actin was from Sigma-Aldrich (A5060). As second antibody we used goat anti-rabbit IgG-HRP (sc-2004; Santa Cruz, CA, USA). Signals were visualized with enhanced chemiluminescence and captured using a scanner (ChemiDoc MP, Bio-Rad, USA) according to the manufacturer's recommendations, the intensities of bands were quantified by digital image analysis software (Quantity One, version 4.6, Bio-RAD, USA).

### Electrophoretic mobility shift assay

Electrophoretic mobility shift assay (EMSA) was performed as described Previously^37^. The DNA-binding activity of NF-κB and AP-1 was detected using a LightShift Chemiluminescent EMSA Kit (Thermo Fisher Scientific, China). RAW264.7 cells in 6-well plates were pretreated with AS1842856 at a concentration of 0.1 μM and 0.5 μM for 12 h and stimulated with 50 ng/mL RANKL for 30 min. Nuclear extracts were prepared using a nuclear and cytoplasmic protein extraction kit according to the manufacturer’s instructions (Beyotime Institute of Biotechnology, Jiangsu, China) and quantified. An equal amount of nuclear extract was incubated with biotin end-labeled duplex DNA and electrophoresed on a 6% polyacrylamide native gel. The AP-1 and NF- κB probes (Beyotime Institute of Biotechnology) used for EMSA, containing the consensus recognition sites for AP-1 and NF-κB, were as follows: AP-1, 5′-CGCTTGATGACTCAGCCGGAA-3′ and NF-κB, 5′-AGTTGAGGGGACTTTCCCAGGC-3′.

### Statistical analysis

All experiments were independently repeated three times. Data were expressed as mean ± standard deviation (SD). One-way analysis of variance (ANOVA) with subsequent Student-Newman-Keuls test was used to determine significant differences in multiple comparisons. All statistical analyses were carried out with SPSS13.0 software (SPSS, Chicago, IL, USA), P < 0.05 was considered statistically significant.

## Additional Information

**How to cite this article**: Tan, P. *et al.* FOXO1 inhibits osteoclastogenesis partially by antagnozing MYC. *Sci. Rep.* **5**, 16835; doi: 10.1038/srep16835 (2015).

## Supplementary Material

Supplementary Information
