# Supplementary material for: FOXO1 inhibits osteoclastogenesis partially by antagnozing MYC
Source: Sci Rep. 2015 Nov 16;5:16835. doi: 10.1038/srep16835 (PMC4645183; doi:10.1038/srep16835)
Supplement: Supplementary Information [file srep16835-s1.pdf]

FOXO1 inhibits osteoclastogenesis partially by antagnozing MYC

Peng Tan, MD[1,a], Hanfeng Guan, MD[1,a], Linka Xie, MD[2], Baoguo Mi, MD[1], Zhong Fang, MD[1], Jing Li, MD[1], Feng Li, MD[1*]

Supplementary information.

Supplemental Figure 1

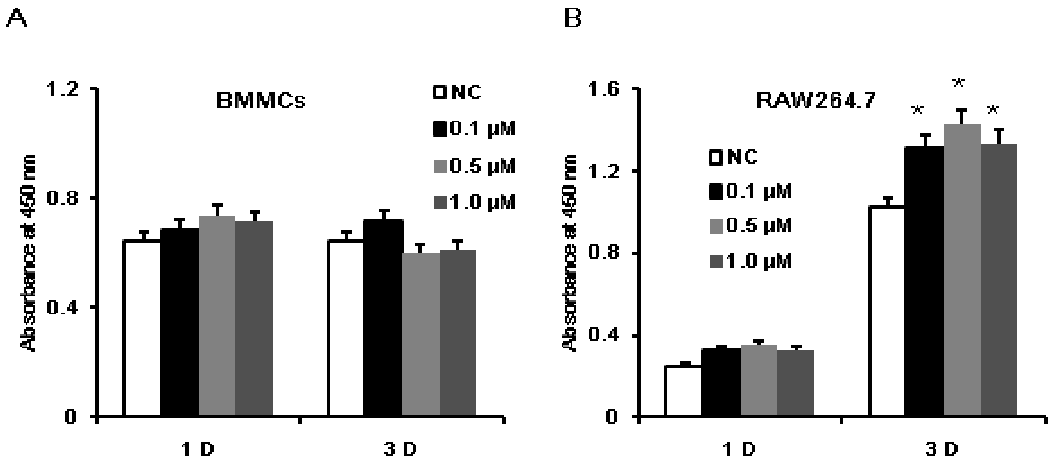

Supplemental Figure 1. FOXO1 inhibition had little influence on the proliferation of BMMCs and RAW264.7 cells. BMMCs and RAW264.7 cells were seeded in 96-well plates at a density of 3000 cells per well and treated with FOXO1 inhibitor AS1842856 at a concentration of 0.1,0.5 and 1.0 μM. Cell viability was measured by CCK8 at the day 1 and 3 after treatment. (A) AS1842856 slightly promoted the proliferation of BMMCs at the concentration of 0.1 μM, however the difference was not statistically significant. (B) AS1842856 mildly promoted the

proliferation of RAW264.7 cells. Data represent the mean ± SD of 3 independent experiments. *P < 0.05 versus control group .

Supplemental Figure 2

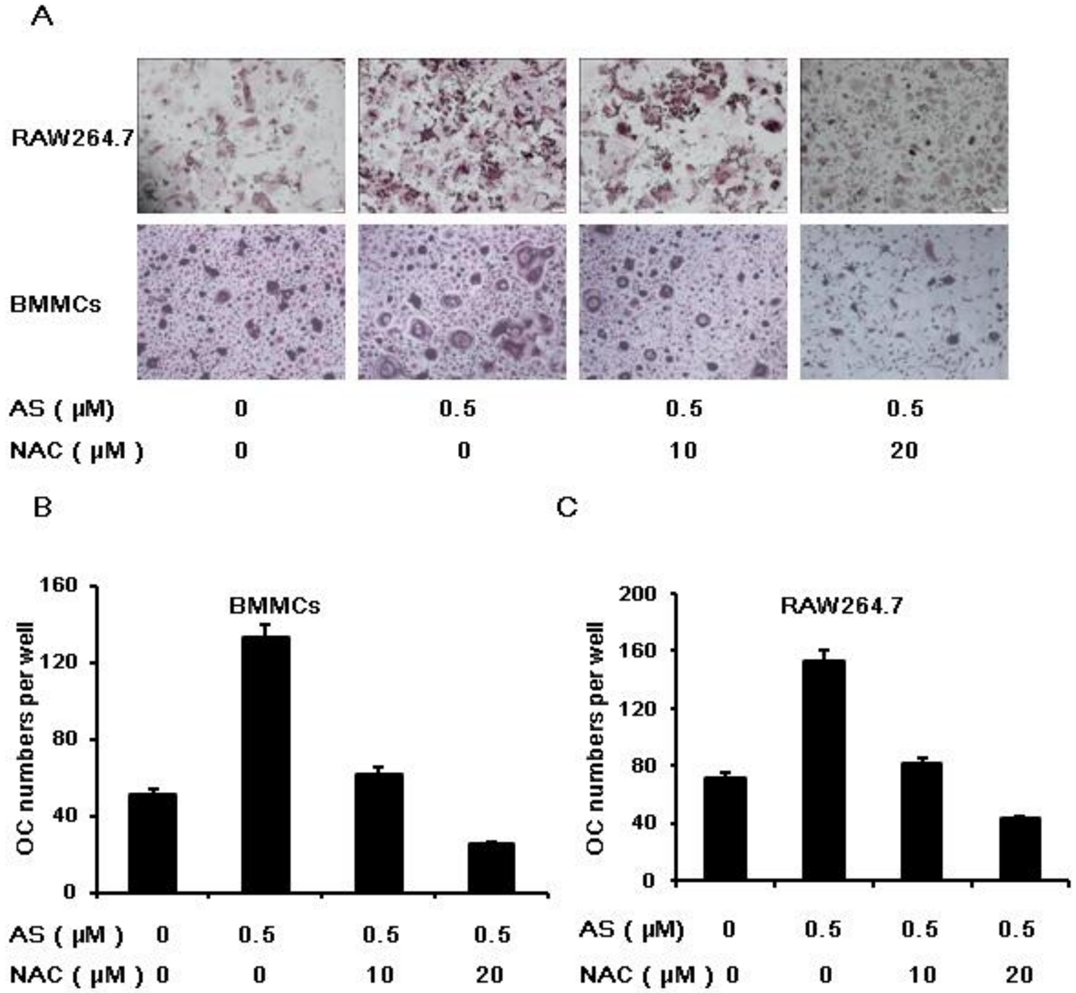

Supplemental Figure 2. N-acetylcysteine (NAC) rescued the osteoclastogenesis promotion effect of FOXO1 inhibition. (A) BMMCs were incubated with M-CSF (30 ng/mL) and RANKL(50 ng/mL); RAW264.7 cells were incubated with RANKL(50 ng/mL), cells were treated with the indicated doses of FOXO1 inhibitor (AS1842856) and NAC. 4 days and 7 days later, RAW264.7 cells and BMMCs cells

were fixed for TRAP staining, respectively. Cells were photographed (original magnification, ×40; A) and the numbers of TRAP-positive multinucleated (>= 3 nuclei) osteoclasts were counted (B, C). Data represent the mean ± SD of 3 independent experiments.
